# Building a Geochemical View of Microbial Salt Tolerance: Halophilic Adaptation of *Marinococcus* in a Natural Magnesium Sulfate Brine

**DOI:** 10.3389/fmicb.2018.00739

**Published:** 2018-04-16

**Authors:** Mark G. Fox-Powell, Charles S. Cockell

**Affiliations:** ^1^UK Centre for Astrobiology, School of Physics and Astronomy, The University of Edinburgh, Edinburgh, United Kingdom; ^2^School of Earth and Environmental Sciences, University of St Andrews, St Andrews, United Kingdom

**Keywords:** brine, habitability, *Marinococcus*, halophile, magnesium sulfate

## Abstract

Current knowledge of life in hypersaline habitats is mostly limited to sodium and chloride-dominated environments. This narrow compositional window does not reflect the diversity of brine environments that exist naturally on Earth and other planetary bodies. Understanding the limits of the microbial biosphere and predicting extraterrestrial habitability demands a systematic effort to characterize ionic specificities of organisms from a representative range of saline habitats. Here, we investigated a strain of *Marinococcus* isolated from the magnesium and sulfate-dominated Basque Lakes (British Columbia, Canada). This organism was the sole isolate obtained after exposure to exceptionally high levels of Mg^2+^ and SO_4_^2-^ ions (2.369 and 2.840 M, respectively), and grew at extremes of ionic strength not normally encountered in Na^+^/Cl^-^ brines (12.141 mol liter^-1^). Its association at the 16S rDNA level with bacterial halophiles suggests that ancestral halophily has allowed it to adapt to a different saline habitat. Growth was demonstrated in media dominated by NaCl, Na_2_SO_4_, MgCl_2_, and MgSO_4_, yet despite this plasticity the strain was still restricted; requiring either Na^+^ or Cl^-^ to maintain short doubling times. Water activity could not explain growth rate differences between media, demonstrating the importance of ionic composition for dictating microbial growth windows. A new framework for understanding growth in brines is required, that accounts for the geochemical history of brines as well as the various stresses that ions impose on microbes. Studies such as this are required to gain a truly universal understanding of the limits of biological ion tolerance.

## Introduction

Hypersaline brines occur naturally on every continent on Earth, as well as on the ocean floor (Wallmann et al., 2002), in the subsurface (Hanor, 1994), and on other planetary bodies (Kargel, 2000; Tosca et al., 2008). Understanding the factors that limit and control microbial growth in brines is therefore crucial to charting the extent of the terrestrial biosphere, as well as predicting likely habitable environments off the Earth.

Knowledge of microbial life in saline systems is largely based on observations of sodium and chloride-dominated environments, which form the majority of hypersaline water bodies on Earth(Hardie, 1985). These are commonly populated by ‘halophilic’ (salt-requiring) or ‘halotolerant’ (salt-tolerant) microbes, and thanks to a focused body of work, we have gained a detailed understanding of the strategies employed by these organisms for coping with molar levels of Na^+^ and Cl^-^ ions (Oren, 2011; Mcgenity and Oren, 2012). High concentrations of salt ions reduce the thermodynamic availability of water (water activity) (Grant, 2004), and because hydration is a fundamental requirement for biomolecular function, reduced water activity is widely recognized as the most significant challenge to life in hypersaline environments (Stevenson et al., 2015).

Yet due to complex ion-specific interactions both with the solvent and directly with biomolecules, there is a strong rationale to expect that brines of different compositions will exert different stresses on microorganisms. Indeed, many studies of both halophiles and their biomolecules have shown that changing the ionic composition of the milieu can influence every aspect of biological function, including protein conformation, enzymatic activity, cellular morphology, growth rate and culture density (e.g., Flannery, 1956; Abram and Gibbons, 1961; Boring et al., 1963; Houchstein and Dalton, 1968; Lanyi, 1974; Madern et al., 2000). Many of these historical studies, whilst they suggest important ion-specific effects, do not account for variations in water activity across different salt solutions, thus making discrimination between ion-specific and hydration-related effects difficult.

More recently, observations of deep-sea hypersaline anoxic basins that contain magnesium chloride brines have shown that chaotropicity (macromolecule-disordering effects), not water activity, defines microbial colonization in the brine: seawater interface (Hallsworth et al., 2007; Yakimov et al., 2015). Chaotropicity is a property of ion combinations, such as Mg^2+^/Cl^-^, that is not exhibited by Na^+^/Cl^-^ brines and which powerfully limits microbial growth (Hallsworth et al., 2003; Ball and Hallsworth, 2015). Furthermore, a previous study has shown that high ionic strength brines rich in divalent ions can limit microbial growth even when water activity is sufficiently permissive (Fox-Powell et al., 2016). Observations such as these raise the questions: how much of what has been learnt about microbial salt adaptation from studying Na^+^/Cl^-^ environments can we apply generally to all brine environments? And how much is ion-specific?

It is currently unclear whether a tendency for generic salt tolerance exists. There is even uncertainty surrounding the words ‘halophilic’ and ‘halotolerant,’ as often no distinction is made between generic salt tolerance and specific adaptation to Na^+^ and Cl^-^. Here, we use the prefix ‘halo’ when discussing specific Na^+^ and Cl^-^ adaptation. Some authors have used similar terms to describe specific tolerance of other salts (e.g., ‘epsophily’ for life in high Mg^2+^/SO_4_^2-^ environments; Crisler et al., 2012, or ‘natronophily’ for life in high Na^+^/HCO_3_^-^ environments; Banciu and Sorokin, 2013), but such terms are not employed here. The significance of these distinctions (including the widely used ‘halophile’) will remain unclear until it is understood how the effects of different ions manifest themselves on an organismal scale, particularly as most brine environments are characterized by dynamic assemblages of salt ions.

A necessary first step in this effort is to systematically study organisms from a diverse range of hypersaline environments, to understand their ion specificities both as a function of and independent from water activity. If halophilic tendencies equip organisms to inhabit non-Na^+^/Cl^-^ brines, then it can be expected that inhabitants of such environments will themselves be halophiles or descendants of halophiles. Alternatively, if halophilic adaptation does not permit growth in other saline environments, it should be expected that brine inhabitants are descended from a variety of clades, regardless of ancestral halophily. Molecular surveys of environments such as the Dead Sea, which contains high concentrations of Mg^2+^ alongside Na^+^ and Cl^-^, have revealed dynamic populations of halophiles whose structures fluctuate in response to changes in ionic composition (Bodaker et al., 2010), whilst the Mg^2+^/SO_4_^2-^ rich Spotted Lake (British Columbia, Canada), and Hot Lake (Washington, Unites States) notably lack the halophilic archaea, which are ubiquitous inhabitants of Na^+^/Cl^-^ brines (Lindemann et al., 2013; Pontefract et al., 2017). Laboratory-produced sulfate brines have also been shown to select for communities lacking typical halophiles, even when such organisms were present in the inoculum (Fox-Powell et al., 2016). Developing a truly universal understanding of the limits of biological ion tolerance requires marrying such community-level studies with in-depth investigations of the physiological limitations and requirements of specific microorganisms.

Here, we used the Basque Lakes, British Columbia, Canada as a natural laboratory to explore biological adaptation to non-Na^+^/Cl^-^ brines. Like neighboring Spotted Lake and Hot Lake, these are a series of Mg^2+^/SO_4_^2-^ dominated hypersaline lakes that in the summer are concentrated to beyond epsomite (MgSO_4_^.^7H_2_O) saturation (Eugster and Hardie, 1978). They bear one of the highest reported divalent: monovalent ion ratios in natural brines and as such are an important analog for divalent ion-rich brines on Mars and elsewhere in the solar system (Foster et al., 2010; Fox-Powell et al., 2016). We report the isolation of a strain of *Marinococcus*, which was capable of growth in extremely high concentrations of Mg^2+^ and SO_4_^2-^. The clustering of this genus with other typical halophiles suggests that the ancestral halophily of *Marinococcus* strain IS_5_B2c has allowed it to adapt to grow under high magnesium sulfate conditions.

## Materials and Methods

### Field Sampling and Geochemical Analyses

**FIGURE 1:**
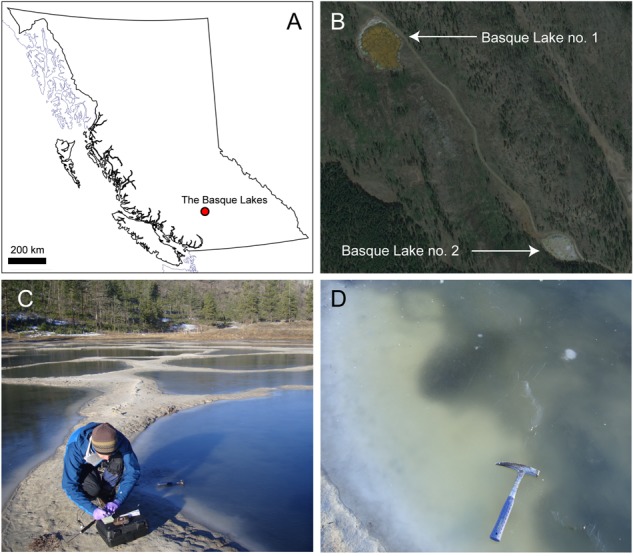
The Basque Lakes sampling locations. **(A)** Lakes within the regional setting of British Columbia, Canada; **(B)** an aerial view of Basque Lakes nos. 1 and 2; **(C)** Basque Lake no. 2 in February 2015; **(D)** individual ice-covered pool within the Basque Lake no. 2 site.

The Basque Lakes are a series of magnesium and sulfate brine lakes, pools and playas in the semi-arid interior of British Columbia, Canada (**Figure 1**). The lakes experience ion concentrations beyond epsomite saturation in the dry summer months (Eugster and Hardie, 1978). Basque Lakes no. 1 (N 50° 36.01 W 121° 21.31) and 2 (N 50° 35.35 W 121° 20.54) were sampled in February 2015. The lakes consisted of numerous smaller ice-covered pools (**Figure 1**). Brine-inundated sediments were sampled from three pools (one from Lake no. 1 and two from Lake no. 2) using sterile 50 ml tubes and spatulas sterilized in 70% ethanol. Tubes were filled to the top to minimize gas phase, sealed with parafilm and kept at 4° C until shipment back to the laboratory. Oxidation/reduction potential, pH and temperature were measured *in situ* using a Myron L Company Ultrameter II and a Fisher Scientific Pt-100 platinum sensor digital thermometer. Lake waters for geochemical analyses were filtered (0.22 μm) and stored in 15 ml tubes for shipment back to the laboratory. Samples intended for cation analysis were acidified with HNO_3_ to a final concentration of 1%.

Major cations were analyzed by Inductively Coupled Plasma Optical Emission Spectroscopy (ICP-OES) at The University of Edinburgh, United Kingdom, using a Perkin Elmer Optima 5300 DV ICP-OES instrument according to manufacturer’s instructions. Major anions were analyzed by Ion Chromatography (IC) at The University of Edinburgh, United Kingdom, using a Dionex DX-120 system according to manufacturer’s instructions.

### Enrichment, Isolation, and Cultivation of a Pure Strain

In order to select for organisms adapted for life in the most extreme concentrations of divalent ions, Basque Lake no. 2 samples were screened using a high ionic strength culture medium (designated IS_5_) designed to maximize ionic strength whilst keeping water activity relatively high (“Control-5” in Fox-Powell et al., 2016). This medium contained (per liter) 67.7 g/0.333 M MgCl_2_⋅6H_2_O, 431.33g/1.75 M MgSO_4_⋅7H_2_O, 213.060 g/1.5 M Na_2_SO_4_, 0.746g/0.010 M KCl, 4g yeast extract (Oxoid), was adjusted to pH 7.0 with a 1 M solution of Tris–HCl and sterilized by autoclaving at 121°C for 15 min. Due to a small amount of precipitate, the medium was sampled and analyzed by ICP-OES and IC as described above. Ionic strength of the completed medium was 12.141 mol liter^-1^.

Fifty milliliter aliquots of IS_5_ medium were inoculated in triplicate with approximately 5 g brine/sediment from each Basque Lake no. 2 sample and shaken at 70 rpm at 30°C for 60 days. Following primary incubation, enrichments were stained with 1× SYBR gold and observed under fluorescence microscopy. Only one sample produced a successful enrichment, which was subsequently transferred (1% v/v) to fresh sterile IS_5_ medium.

A single pure strain (designated IS_5_B2c) was isolated in IS_5_ medium by three rounds of serial dilution-to-extinction. Briefly, a 10-fold dilution series of cells was established, spanning a range of dilutions that encompassed and exceeded the cell density (enumerated by direct counts under fluorescence microscopy). These dilutions were incubated as before and monitored for growth. The highest dilution to exhibit growth, which was founded by a population of approximately 1 cell ml^-1^, was then used to initiate a second round of dilutions. This process was repeated a total of three times. The highest dilution to exhibit growth from the third round was selected for future experiments. Cells were harvested by filtration (0.22 μm) and frozen for DNA extraction and confirmation of strain purity.

Due to extremely slow growth in IS_5_ medium, strain IS_5_B2c was routinely cultured in a medium simulating the relative ionic abundances of Basque Lake no. 2 (hereafter referred to as BL medium). BL medium was less concentrated, possessing a lower ionic strength than IS_5_ medium (∼8.7 mol liter^-1^), and preliminary experiments showed dramatic increases in growth rates compared to IS_5_ medium. BL medium contained (per liter): 4.11 g/0.041 M KHCO_3_, 1.521 g/0.020 M KCl, 11.379 g/0.056 M MgCl_2_⋅6H_2_O, 8.983 g/0.154 M NaCl, 509.775 g /2.068 M MgSO_4_⋅7H_2_O, 4 g yeast extract (Oxoid), 3 g casamino acids (Difco), was adjusted to pH 7.0 with a 1 M solution of Tris–HCl and sterilized by autoclaving at 121°C for 15 min.

### DNA Extraction, Sequencing, and Phylogenetic Analysis

Strain IS_5_B2c was grown to stationary phase in BL medium, harvested by filtration onto sterile 0.22 μm-pore 25 mm diameter polycarbonate filters (Merck Millipore) and DNA was extracted using a modified phenol:chloroform:isoamyl alcohol protocol as described in Urakawa et al. (2010). DNA was interrogated by polymerase chain reaction (PCR) using the primers 27F (5′-AGAGTTTGATCMTGGCTCAG-3′) and 1389R (5′-ACGGGCGGTGTGTACAAG-3′) which target bacterial 16S ribosomal RNA (Cameron et al., 2012). Each individual 25 μl PCR reaction contained 1 μl template, 0.4 μM of the relevant forward and reverse primer, 200 μM dNTPs, 1.5 mM MgCl_2_, 1× PCR buffer and 1 unit *Taq* polymerase (Invitrogen). Reactions were treated at 95°C for 5 min and then subject to 25 cycles of: melting at 95°C for 60 s, annealing at 55°C for 60 s and extension at 72°C for 90 s, and finished with a final extension step at 72°C for 10 min. Bacterial PCR products were purified using the Qiagen Amplicon Purification Kit according to manufacturer’s instructions, and sequenced on the Sanger ABI 3730 XL platform at Edinburgh Genomics, The University of Edinburgh, in both forward and reverse directions.

Sequence processing, trimming and contig assembly was performed using the DNA Baser software. First, primer sequences were removed from both complementary sequences. Next, the sequences were aligned for contig assembly and low-quality ends and ambiguous bases were removed. The final consensus sequence was exported for phylogenetic analysis and aligned with the NCBI database using the BLASTn tool. Phylogenetic analysis was performed using the CLUSTAL_W program in the MEGA v. 7 software suite. A phylogenetic tree was constructed using the neighbor joining approach (Saitou and Nei, 1987), with *Alicyclobacillus acidocaldarius* used as the outgroup (Wang et al., 2009). The topology of the tree was evaluated by the bootstrap resampling method based on 1000 replicates.

### Growth Response Quantification

Growth of strain IS_5_B2c was quantified by measuring increase in optical density at 600 nm (OD_600_) in 96 well plates in a Biotek Synergy 2 microplate reader. Doubling times were calculated from the exponential growth portion of OD_600_ curves. The pH-defined growth range of strain IS_5_B2c was investigated by recording OD_600_ increase in a series of BL media buffered in 0.5 pH unit increments between pH 3.0 and pH 9.0. Levels of pH above 7.5 were achieved by the addition of Tris; levels below pH 6.5 were adjusted with HCl. Temperature ranges were established by recording OD_600_ increase in BL medium (pH 7.0) at increments of increasing temperature from 4 to 45°C. The NaCl requirement (halophily) of strain IS_5_B2c was established in a series of NaCl media, in which the most concentrated contained (per liter) 292.2 g/5.0 M NaCl and concentration was decreased in either 0.5 or 0.25 M increments. NaCl media also contained (per liter): 0.746 g/0.010 M KCl, 4 g yeast extract (Oxoid) and 3 g casamino acids (Difco).

The ionic requirements of strain IS_5_B2c were tested by systematically comparing growth rates between media that contained 2 M of each of four ions (Mg^2+^, Na^+^, SO_4_^2-^, and Cl^-^) in all possible cation-anion pairings. Apart from the main dissolved salt, the media contained (per liter) 0.746 g/0.010 M KCl, 4 g yeast extract (Oxoid) and 3 g casamino acids (Difco).

### Water Activity Measurement

Water activity was quantified at 30°C using a Rotronic HP23-AW water activity meter in AWE mode, calibrated to five points (0.325, 0.595, 0.755, 0.845, and 0.935) using saturated salt solution standards (MgCl_2_, NH_4_NO_3_, NaCl, KCl, and KH_2_PO_4_, respectively) prepared as described by Winston and Bates (1960).

## Results

### The Basque Lakes

At time of sampling, the waters of both Basque Lake no. 1 and Basque Lake no. 2 were in a relatively dilute phase likely due to increased rain and snowmelt input and lower evaporation rates in winter. The three sampled pools (distributed between Lakes nos. 1 and 2) possessed ionic strengths between 0.498 and 1.038 mol liter^-1^, magnesium/sodium ratios between 1.50 and 3.56 and sulfate/chloride ratios between 28.31 and 86.92 (**Table 1**).

**Table 1:** Ionic composition and physicochemical parameters of the Basque Lakes sample sites.

	Basque 1	Basque 2b	Basque 2c
K^+^	0.094	0.184	0.184
Na^+^	0.645	1.543	1.623
Mg^2+^	2.105	2.449	6.113
Ca^2+^	0.172	0.196	0.171
SO_4_^2-^	9.051	11.950	23.392
Cl^-^	0.100	0.156	0.099
Mg: Na	3.084	1.502	3.562
SO_4_: Cl	33.301	28.306	86.917
Ionic strength/ mol liter^-1^	0.387	0.498	1.038
Temperature/°C	0.9	0.9	2.9
pH	6.23	6.26	5.8
Conductivity/mS	13.9	28.6	17.83
ORP (mV)	–150	*nd*	–24

### *Marinococcus* Strain IS_5_B2c

**FIGURE 2:**
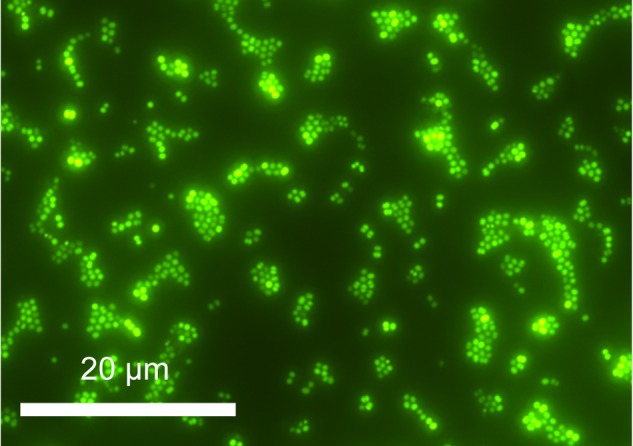
Cells of strain IS_5_B2c stained with SYBR gold, mounted on polycarbonate 0.22 μm filter and imaged under epifluorescence microscopy.

A single isolate, originating from Basque Lake no. 2 and designated strain IS_5_B2c, was obtained by serial dilution-to-extinction. Growth in the high ionic strength (12.141 mol liter^-1^) enrichment medium was slow; taking around 14 days for cell numbers to increase from inoculum values in microscopic observations. Cells of strain IS_5_B2c were 1–2 μm diameter coccoids (**Figure 2**), which when grown to high density in the routine BL medium exhibited a noticeably visible orange-yellow pigmentation.

**FIGURE 3:**
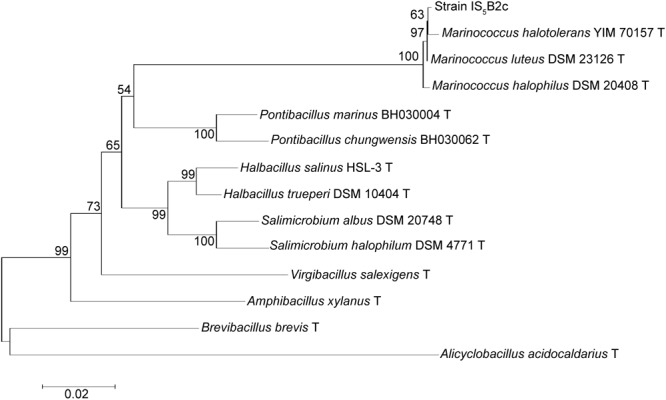
Phylogenetic association of strain IS_5_B2c with the *Marinococcus* genus and other related taxa, based on partial 16S rDNA sequence. Numbers on branch nodes are percentage bootstrap values from 1000 resampling efforts. The root was the 16S rDNA sequence from *Alicyclobacillus acidocaldarius* DSM 446. Scale bar represents number of base substitutions per site.

A partial 16S rDNA sequence was obtained, covering 1,117 base pairs. At the 16S rDNA level, strain IS_5_B2c clustered most closely with the moderately halophilic, aerobic Gram-positive bacterial genus *Marinococcus*, sharing >99% identity with all other described species in the genus (**Figure 3**). BLAST analysis revealed the closest database match at 16S rDNA level to be from a bacterial culture originating from Sehline Sebkha salt lake, Tunisia, which also clustered in the *Marinococcus* genus (Hedi et al., 2014).

**FIGURE 4:**
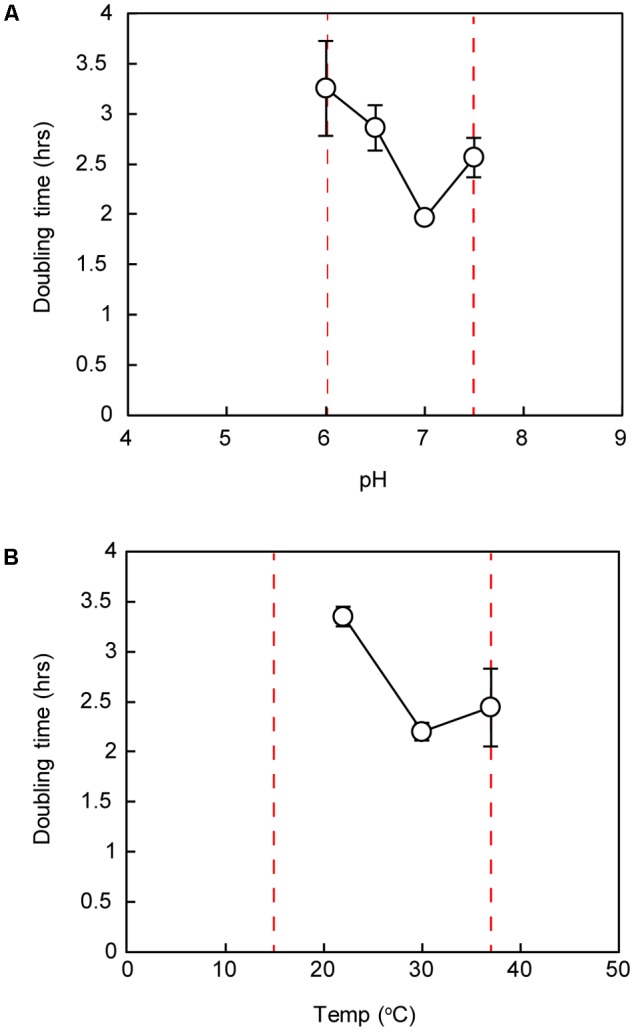
Doubling time of strain IS_5_B2c under ranges of pH **(A)** and temperature **(B)**. Dashed red lines represent maximum and minimum growth limits. Bars represent ± 1 standard error of triplicate experiments.

Similar to previously described *Marinococcus* strains, strain IS_5_B2c grew optimally at pH 7.0, with short doubling times also recorded at pH 6.5 and 7.5. No growth was observed below pH 6.0 or above pH 7.5 (**Figure 4**). Strain IS_5_B2c grew optimally at a similar temperature to other *Marinococcus* isolates, which have temperature optima around 30°C (Novitsky and Kushner, 1976; Van Hao et al., 1984; Li et al., 2005; Wang et al., 2009) and was capable of slow growth at 15° C (growth rate not quantified), but not at 4°C, or at 45°C (**Figure 4**).

### Growth of Strain IS_5_B2c in Varied Ionic Regimes

**FIGURE 5:**
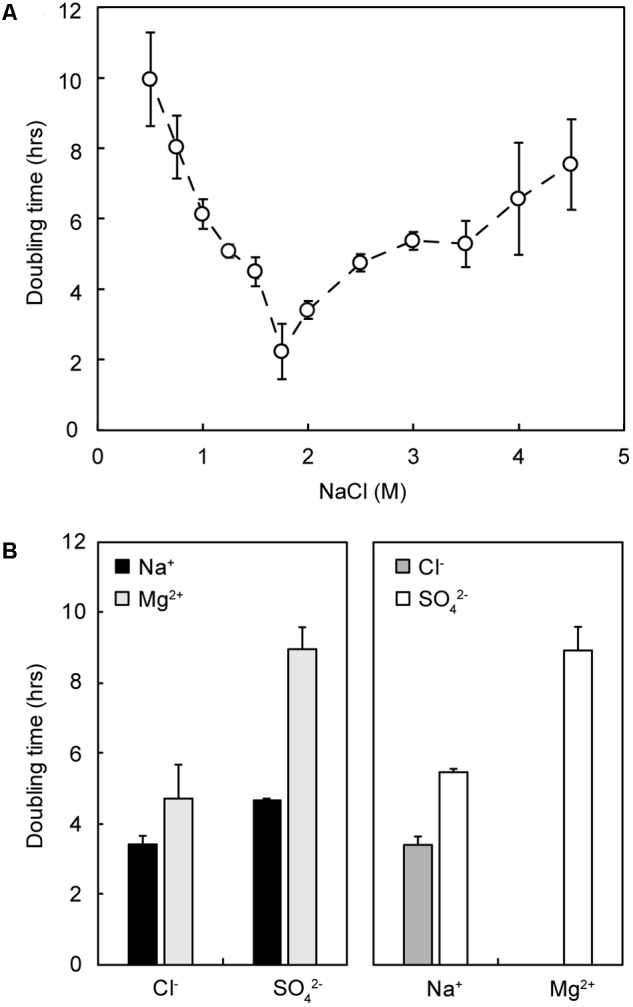
Growth response of strain IS_5_B2c to different pure salt media. **(A)** Growth rate (doubling time) as a function of varied NaCl concentration; **(B)** growth rate in media comprising 2 M of either each anion (Left) or cation (Right), paired with both corresponding counterions. Error bars represent ± 1 standard error of triplicate experiments.

NaCl requirements for strain IS_5_B2c were similar to other members of the genus; growth was demonstrated over a NaCl concentration range of 0.5–4.0 M (approximately 3–24%), with the fastest growth rate around 1.75–2 M (**Figure 5**). No growth was observed at 5 M or at 0.25 M NaCl. The doubling time of *Marinococcus* strain IS_5_B2c was approximately the same in BL medium (dominated by Mg^2+^ and SO_4_^2^) as in the optimum concentration of pure NaCl.

Strain IS_5_B2c showed remarkable plasticity with its ionic requirements, growing in media where the main salts were NaCl, Na_2_SO_4_, MgCl_2_, or MgSO_4_ (**Figure 5**). Growth rates across the different media compositions varied considerably. The most rapid growth was measured in 2 M Na^+^/Cl^-^, and the slowest in 2 M Mg^2+^/SO_4_^2-^⋅ 2 M SO_4_^2-^ (paired with Na^+^) supported a comparable growth rate to 2 M Cl^-^ (paired with Mg^2+^). Doubling times in 2 M Na^+^ when paired with SO_4_^2-^ were slightly longer (**Figure 5**).

**FIGURE 6:**
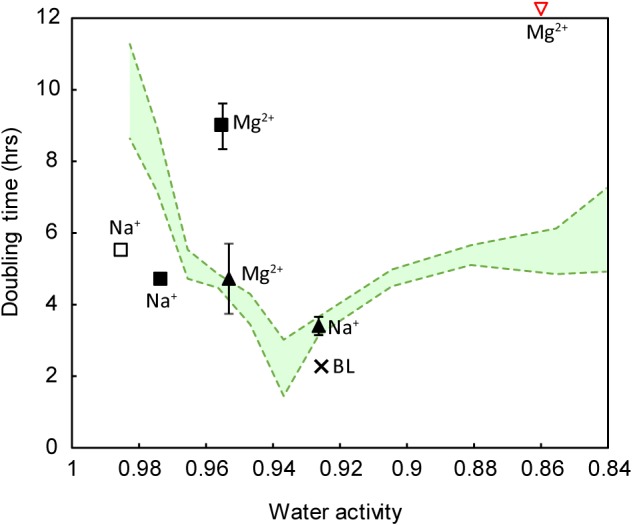
Growth response of strain IS_5_B2c to varied ionic regimes, as a function of water activity. Pure salt media (including all possible cation-anion pairings) are the same as those reported in **Figure 5**. Green shaded area represents the water activity response curve (±1 standard error) of the strain when grown in pure NaCl media. Square symbols indicate sulfate salts, triangles indicate chloride salts. Filled symbols indicate media containing either 2 M of the anion or 2 M of both anion and cation (in the case of salts with a 1: 1 stoichiometry). Empty symbols indicate media containing 2 M of the cation only. BL = BL medium, plotted for reference. Note that 2 M Mg^2+^ paired with chloride inhibited all growth (inverted red triangle). Error bars represent ± 1 standard error of triplicate experiments.

To attempt to explain the different growth rates, we measured water activity in all growth media. We found that when grown in NaCl, strain IS_5_B2c had a water activity optimum of approximately 0.940 a_w_, with similar growth rates obtained between 0.950 a_w_ and 0.920 a_w_ (**Figure 6**). Higher and lower water activities produced progressively longer doubling times. Synthetic Basque Lakes medium (BL medium), at 0.925 a_w_, supported rapid doubling times in agreement with the optimum water activity range in NaCl. Water activity varied considerably across the other salt media, despite sharing absolute concentrations of specific ions. Most ion pairings produced doubling times that fell on or near the NaCl water activity growth response curve, with the notable exception of 2 M Mg^2+^ paired with Cl^-^ (which prohibited all growth) and 2 M Mg^2+^/SO_4_^2-^, which had a water activity near the optimum value yet a growth rate close to the slowest recorded here (**Figure 6**).

## Discussion

### The Basque Lakes

The Basque Lakes, British Columbia are part of a series of hypersaline playas in western North America that exhibit unusual chemistries (Renaut, 1990). The Basque Lakes are particularly unique for not simply containing Mg^2+^ and SO_4_^2-^ as major ions, but for being dominated by them, reflected in high Mg^2+^: Na^+^ ratios and even higher SO_4_^2-^: Cl^-^ ratios (**Table 1**). This water chemistry is similar to that found in nearby Spotted Lake (Pontefract et al., 2017). Such environments provide valuable natural laboratories for understanding microbial salt tolerance, and due to their high divalent : monovalent ion ratios make compelling analogs for sulfate brines on ancient Mars, which likely possessed divalent ion content highly atypical of terrestrial chloride brines (Vaniman et al., 2004; Tosca et al., 2011). Indeed, sulfates (Mg, Fe, and Ca) make up the majority of martian salt minerals both in evaporite outcrops (Bibring et al., 2006; Hynek et al., 2015), and in the globally distributed dust (Yen et al., 2005), recording the widespread presence of sulfate-rich brines during a key transitionary phase of martian surface evolution. It follows that properly understanding how martian habitability has changed through time requires knowledge of how life adapts to cope with highly saline sulfate-rich environments. Thus, isolates from environments such as the Basque Lakes can help inform what establishes boundaries to biologically permissive environments on other planetary bodies, whether that be indigenous life or less speculatively, microorganisms transferred into these environments by robotic or human missions.

### *Marinococcus* Strain IS_5_B2c: A Magnesium and Sulfate-Loving Halophile

A strain of *Marinococcus*, designated IS_5_B2c, was isolated from the Basque Lakes using a high ionic strength growth medium. The successful cultivation of strain IS_5_B2c at an ionic strength of 12.141 mol liter^-1^ in IS_5_ medium, with a Mg^2+^ concentration of 2.369 M and a SO_4_^2-^ concentration of 2.840 M represents both the highest documented ionic strength and the highest co-occurring concentrations of Mg^2+^ and SO_4_^2-^ directly demonstrated to be tolerated by a microorganism. Growth at these levels is remarkable, particularly considering that high ionic strength (>10 mol liter^-1^) can interact with other stressors to powerfully limit microbial growth, precluding halophiles that tolerate more extreme levels of low water activity (Fox-Powell et al., 2016).

Similar growth rates recorded in BL medium (dominated by Mg^2+^ and SO_4_^2-^ with Na^+^ and Cl^-^ approximately an order of magnitude lower in concentration) and in the optimum concentration of pure NaCl (**Figure 5**) is a likely sign of adaptation to its source environment. The replacement of Na^+^/Cl^-^ with Mg^2+^/SO_4_^2-^ as the dominant ions with no negative effect on growth hints at a set of evolutionary adaptations in the Basque Lakes which could supply a host of valuable information on the ion specificity of salt stress.

The *Marinococcus* genus is classified at the high end of moderate halophily; members typically grow optimally over the range 10–15% dissolved NaCl (approximately 1.8–2.6 M), but some can tolerate higher concentrations (up to 25%) (Li et al., 2005; Rice et al., 2009). The placing of strain IS_5_B2c within the *Marinococcus* genus, and this genus’s close phylogenetic association with other halophilic genera (including *Pontibacillus, Halobacillus*, and *Salimicrobium*) is a clear demonstration that halophilic (NaCl-loving) organisms are not precluded from hypersaline environments of fundamentally different compositions. It is likely that physicochemical stressors common to all hypersaline environments, such as low water activity, ensure that halophilic organisms already equipped to deal with them have a competitive advantage over those that are not.

However, other strains of *Marinococcus* are associated with brine compositions that suggest deviations from traditional halophily. The closest database match for strain IS_5_B2c was an isolate from Sehline Sebkha salt lake, Tunisia, which is dominated by Na^+^ and Cl^-^ but with significant concentrations of Mg^2+^ and SO_4_^2-^ as secondary ions (Hedi et al., 2014). *Marinococcus halotolerans*, isolated from hypersaline soils in China, was initially enriched in a medium containing 250 gL^-1^ MgCl_2_.6H_2_O (1.23 M MgCl_2_), with relatively low concentrations of NaCl (2 g L^-1^; 33 mM) (Li et al., 2005). The variety of saline conditions tolerated by this organism are remarkable; the authors report similar growth responses across wide concentration ranges of NaCl, KCl, and MgCl_2_, which is rare for halophilic or halotolerant organisms which usually require NaCl to some degree (Boring et al., 1963; Javor, 1984; Ventosa et al., 1998; Crisler et al., 2012). This plasticity is also reflected in strain IS_5_B2c, as discussed below. Enrichments done in high levels of Mg^2+^ by Li et al. (2005) and in the current study both resulted in the culture of a strain of *Marinococcus*, despite the large degree of geographical separation between the source environments. Due to their ability to tolerate a seeming wide variety of ionic compositions, including those of extreme ionic strength, species within the *Marinococcus* genus make compelling model organisms for understanding the capacity for life in hypersaline environments that differ significantly from typical Na^+^/Cl^-^ brines.

### Beyond Water Activity: Ion Specificities of *Marinococcus* Strain IS_5_B2c

Developing a universal understanding of microbial ion tolerance requires systematic assessments of the ionic requirements of organisms from a range of hypersaline environments. It is only with an appreciation of how ionic composition defines growth in the context of an organism’s phylogenetic associations that we can begin to discriminate between generic salt adaptation tendencies and ion-specific effects. Historical efforts to investigate the ionic dependencies of halophiles produced results that frustrated attempts to generalize; organism-specific idiosyncrasies seemed to be the rule (e.g., Javor, 1984; Ventosa et al., 1998). Consistencies that did emerge were that all halophiles require Na^+^ to some degree, and that the requirement for Na^+^ and Cl^-^ ions could be lowered somewhat through substitution with other solutes (including organic solutes such as glucose or glycerol) (Adams et al., 1987). However, these studies lacked systematic consideration of other powerful growth-mediating physicochemical parameters, such as water activity, which can vary significantly across different brines.

Water activity is a measure of the thermodynamic availability of water, expressed relative to pure water at the same temperature and pressure. The degree to which water activity is depressed in an aqueous system varies with the solute. For example, 2 M solutions of NaCl and MgSO_4_ have respective water activities of approximately 0.93 a_w_ and 0.96 a_w_, which is greater than the difference between freshwater and seawater. Given that water activity is a powerful mediator of microbial growth (Stevenson et al., 2015), any assessment of the effects of ionic composition on microbial growth requires consideration of this parameter.

Strain IS_5_B2c was therefore subjected to suite of growth trials in which the main dissolved salt was varied systematically (by concentration), and water activity was measured across all media. When strain IS_5_B2c was grown in different pure salt media, growth response varied considerably, despite the media sharing absolute concentrations of ions; as has been observed in many strains (Abram and Gibbons, 1961; Boring et al., 1963; Javor, 1984; Ventosa et al., 1998). It is tempting to interpret this as preferences for certain ions; for example in both 2 M Cl^-^ and 2 M SO_4_^2-^, doubling times were shorter if the cation was Na^+^ than Mg^2+^ (**Figure 6**), inviting the conclusion that strain IS_5_B2c has a preference for Na^+^ over Mg^2+^.

However, considering water activity alongside ionic composition explained some of strain IS_5_B2c’s apparent ionic preferences. An apparent preference for Na^+^ over Mg^2+^ when paired with 2 M Cl^-^ was satisfactorily explained by a higher water activity in the Mg^2+^/Cl^-^ media (which had a concentration of 1 M); meaning that it lay just outside the organism’s optimum range (**Figure 6**). When the 2 M Cl^-^ (with Mg^2+^) growth rates were compared with NaCl media at approximately the same water activity, doubling times were in excellent agreement despite differences in absolute Cl^-^ concentration (**Figure 6**). This is a clear demonstration that if ionic preferences are to be understood, physicochemical factors such as water activity must be accounted for.

Water activity alone cannot be used to predict doubling time, however, otherwise all doubling times would have fallen somewhere on the optimum curve, which was not the case. Clearly, ion-specific effects were important. 2 M Mg^2+^ paired with Cl^-^ inhibited all growth, despite possessing a water activity that was demonstrably permissive in NaCl. This is likely due to the highly chaotropic activity of MgCl_2_ at these concentrations (2 M) (Cray et al., 2013). It has previously been shown that chaotropic solutes such as MgCl_2_ can impose stresses on microorganisms even when water activity is at permissive levels (Hallsworth et al., 2003), and currently biological activity of any kind above 2.3 M MgCl_2_ has not been documented (Hallsworth et al., 2007; Yakimov et al., 2015). Both Na_2_SO_4_ media produced doubling times slightly faster than that predicted from water activity alone, suggesting a preference for sulfate under more dilute conditions.

Perhaps most striking are the notably long doubling times recorded in 2 M MgSO_4_, indicating a much slower growth rate than that predicted by the water activity curve in NaCl (**Figure 6**). This is despite being isolated from an environment characterized by high levels of these ions. Again, this cannot be explained by high ionic strength or some toxic effect of either Mg^2+^ or SO_4_^2-^, as the strain grew readily in BL medium (dominated by Mg^2+^ and SO_4_^2-^ with an ionic strength ∼0.5 mol liter^-1^ higher than the pure MgSO_4_ medium), at the shortest doubling time achieved for the strain. It is important to note, however, that BL medium also contained Na^+^ and Cl^-^ as secondary ions.

When both Na^+^ and Cl^-^ were omitted from the growth media, growth was dramatically retarded. Surprisingly, it was not a requirement for Na^+^ and Cl^-^ in combination that defined this limitation, as the organism would grow well in media where one of either Cl^-^ or Na^+^ had been removed (for instance, in 1 M MgCl_2_ or 1 and 2 M Na_2_SO_4_). Rather, if either of these ions were present in appreciable quantities, it was sufficient to stimulate growth. This is remarkable, as cations and anions are handled by wholly different cellular pathways and thus it is highly implausible that there could be some active cellular process that could readily substitute Na^+^ for Cl^-^. It could instead be due to some affinity for specific ions in the structure of the organism’s biomolecules, as is observed in the halophilic archaea, and also in the membrane proteins of some bacterial halophiles (Oren et al., 2005).

The Haloarchaea have evolved to accept molar quantities of K^+^ (in place of Na^+^) and Cl^-^ into the cell, and their intracellular machinery must be structurally modified to function in the presence of these ions (Soppa, 2006). The observed changes mostly decrease hydrophobicity and increase protein affinity for water molecules, both of which seem to be approaches to mitigate low water activity stress (Tadeo et al., 2009). However, enzymes from these organisms are shown to have ion specificities beyond a simple need for hydration, and function optimally only if K^+^ and Cl^-^ are provided (Ortega et al., 2011; Karan et al., 2012). A narrow window of ion-specificity in the Haloarchaea might explain their absence from Mg^2+^/SO_4_^2-^ environments like Spotted Lake, British Columbia (Pontefract et al., 2017), and sulfate brine enrichments (Fox-Powell et al., 2016), as well as the loss of Haloarchaeal dominance in the Dead Sea following evaporative concentration of Mg^2+^ (Bodaker et al., 2010).

Due to an ostensible lack of these proteome modifications in halophiles that maintain low cytoplasmic ion concentrations, such as most halophilic bacteria (including the *Marinococcus* genus; Louis and Galinski, 1997), it could be predicted that such organisms are more plastic with their ionic requirements, responding predominately to changes in water activity. However, studies have shown that even bacterial halophiles still bear some of the signatures of ‘salt-in’ Haloarchaeal proteomes, particularly in their membrane bound proteins which are exposed to the full salinity of the external milieu (Oren et al., 2005). Some specificity should therefore be expected, even in halophiles whose cytoplasmic salt ion content never rises beyond that of non-halophiles. Our data support this prediction, as growth appeared to be controlled predominately by hydration (water activity) effects as long as either Na^+^ or Cl^-^ were available; when both of these ions were removed, growth was dramatically slowed (**Figure 6**).

Future work should focus on determining the mechanisms employed by strain IS_5_B2c for coping with extremely high Mg^2+^ and SO_4_^2-^ concentrations. In particular, changes in metabolic pathways and substrate utilization in strain IS_5_B2c under different saline conditions is important follow-on work. As Mg^2+^ concentrations in the Dead Sea became elevated, archaeal CorA magnesium channels became enriched 11-fold (Bodaker et al., 2010). It was assumed this was a resistance mechanism in the halophilic archaea to cope with the changing composition, and if so implies that the typically ‘salt-in’ Haloarchaea were actively excluding the divalent cation. If this energy-demanding process is the only approach available to organisms in high Mg^2+^ environments then that may well be a determining factor for brine habitability.

### Toward a Geochemical View of Salt Ion Tolerance: Lessons Learned From a Model Strain

Most knowledge of how microorganisms grow in hypersaline environments is drawn from studies of sodium and chloride-rich brines. Whilst this approach has served us well in understanding the distribution and activity of halophiles in the most dominant brine type on Earth, it has limited our ability to predict the extent of microbial habitat into other brine types, including those that exist in evaporites in the deep subsurface (e.g., Payler et al., unpublished) or on other planetary bodies (Fox-Powell et al., 2016).

A future goal of mapping the limits of life in high salt is to be able to analyze the composition of any brine and make informed, accurate predictions about habitability. Trade-offs almost certainly exist, as coping with high levels of salts is energetically expensive, meaning that certain ionic compositions might preclude low-energy metabolisms (Oren, 2011). Additionally, if protein structural changes always bear ion specificity, adaptations required to cope with one major ion might always decrease tolerance of another. These questions and others can be answered with systematic efforts to characterize further organisms from diverse brine types. It is notable that even in a brine fundamentally different from typical Na^+^/Cl^-^ environments, halophiles such as *Marinococcus* can thrive, demonstrating that halophily does bestow aspects of a generic salt tolerance. The predictive power of water activity for growth rate was demonstrated across some pure salts, suggesting a limited ionic plasticity of this form of halophily. However, ionic specificity remained; in the absence of Na^+^ and Cl^-^, water activity no longer satisfactorily predicted growth rate.

This finding raises many questions about what might be required on a molecular level to develop true independence from sodium and/or chloride, and whether this has occurred on Earth. It is an open question whether organisms from hypersaline environments can be fully composition-independent and respond only to water activity, or if some ion-specificity will always occur. If this requirement for either Na^+^ or Cl^-^ is indeed a residual trait from strain IS_5_B2c’s halophilic ancestors, would inhabitants of Mg^2+^/SO_4_^2-^ brines that don’t share this evolutionary history exhibit a different set of requirements? Such organisms, should they exist, should be the target of future cultivation efforts.

## Author Contributions

CC and MF-P obtained the samples and contributed equally to experimental design and interpretation of results. MF-P conducted laboratory work and wrote the manuscript. CC contributed text, reviewed and edited the manuscript.

## Conflict of Interest Statement

The authors declare that the research was conducted in the absence of any commercial or financial relationships that could be construed as a potential conflict of interest.

## References

[B1] Abram D. Gibbons N. E. (1961). The effect of chlorides of monovalent cations, urea, detergents, and heat on morphology and the turbidity of suspensions of red halophilic bacteria. *Can. J. Microbiol.* 7 741–750. 10.1139/m61-088 13859031

[B2] Adams R. Bygraves J. Kogut M. Russell D. N. J. (1987). The role of osmotic effects in haloadaptation of *Vibrio costicola*. *J. Gen. Microbiol.* 133 1861–1861. 10.1099/00221287-133-7-1861 3668500

[B3] Ball P. Hallsworth J. (2015). Water structure and chaotropicity: their uses, abuses and implications for biology. *Phys. Chem. Chem. Phys.* 17 8297–8305. 10.1039/C4CP04564E 25628033

[B4] Banciu H. L. Sorokin D. Y. (2013). “Adaptation in haloalkaliphiles and natronophilic bacteria,” in *Polyextremophiles: Life Under Multiple Forms of Stress* eds Seckbach J. Oren A. Stan-lotter H. (Dordrecht: Springer) 121–178. 10.1007/978-94-007-6488-0_5

[B5] Bibring J.-P. Langevin Y. Mustard J. F. Poulet F. Arvidson R. Gendrin A. (2006). Global mineralogical and aqueous mars history derived from omega/mars express data. *Science* 312 400–404. 10.1126/science.1122659 16627738

[B6] Bodaker I. Sharon I. Suzuki M. T. Feingersch R. Shmoish M. Andreishcheva E. (2010). Comparative community genomics in the Dead Sea: an increasingly extreme environment. *ISME J.* 4 399–407. 10.1038/ismej.2009.141 20033072

[B7] Boring J. Kushner D. J. Gibbons N. E. (1963). Specificity of the salt requirement of *Halobacterium cutirubrum*. *Can. J. Microbiol.* 9 143–154. 10.1139/m63-020

[B8] Cameron K. A. Hodson A. J. Osborn A. M. (2012). Structure and diversity of bacterial, eukaryotic and archaeal communities in glacial cryoconite holes from the Arctic and the Antarctic. *FEMS Microbiol. Ecol.* 82 254–267. 10.1111/j.1574-6941.2011.01277.x 22168226

[B9] Cray J. A. Russell J. T. Timson D. J. Singhal R. S. Hallsworth J. E. (2013). A universal measure of chaotropicity and kosmotropicity. *Environ. Microbiol.* 15 287–296. 10.1111/1462-2920.12018 23145833

[B10] Crisler J. D. Newville T. M. Chen F. Clark B. C. Schneegurt M. A. (2012). Bacterial growth at the high concentrations of magnesium sulfate found in Martian soils. *Astrobiology* 12 98–106. 10.1089/ast.2011.0720 22248384 PMC3277918

[B11] Eugster H. P. Hardie L. A. (1978). “Saline lakes,” in *Lakes: Chemistry, Geology, Physics* ed. Lerman A. (New York, NY: Springer) 237–293. 10.1007/978-1-4757-1152-3_8

[B12] Flannery W. L. (1956). Current status of knowledge of halophilic bacteria. *Bacteriol. Rev.* 20 49–66. 13341820 10.1128/br.20.2.49-66.1956 PMC180849

[B13] Foster I. S. King P. L. Hyde B. C. Southam G. (2010). Characterization of halophiles in natural MgSO_4_ salts and laboratory enrichment samples: astrobiological implications for Mars. *Planet. Space Sci.* 58 599–615. 10.1016/j.pss.2009.08.009

[B14] Fox-Powell M. G. Hallsworth J. E. Cousins C. R. Cockell C. S. (2016). Ionic strength is a barrier to the habitability of Mars. *Astrobiology* 16 427–442. 10.1089/ast.2015.1432 27213516

[B15] Grant W. D. (2004). Life at low water activity. *Philos. Trans. R. Soc. B Biol. Sci.* 359 1249–1267. 10.1098/rstb.2004.1502 15306380 PMC1693405

[B16] Hallsworth J. E. Heim S. Timmis K. N. (2003). Chaotropic solutes cause water stress in *Pseudomonas putida*. *Environ. Microbiol.* 5 1270–1280. 10.1111/j.1462-2920.2003.00478.x 14641573

[B17] Hallsworth J. E. Yakimov M. M. Golyshin P. N. Gillion J. L. M. D’Auria G. De Lima Alves F. (2007). Limits of life in MgCl2-containing environments: chaotropicity defines the window. *Environ. Microbiol.* 9 801–813. 10.1111/j.1462-2920.2006.01212.x 17298378

[B18] Hanor J. S. (1994). Origin of saline fluids in sedimentary basins. *Geol. Soc. London Spec. Publ.* 78 151–174. 10.1144/GSL.SP.1994.078.01.13

[B19] Hardie L. A. (1985). Evaporites: marine or non-marine? *Am. J. Sci.* 285 667–672. 10.2475/ajs.285.7.661

[B20] Hedi A. Essghaier B. Cayol J.-L. Fardeau M.-L. Sadfi N. (2014). Prokaryotic biodiversity of halophilic microorganisms isolated from Sehline Sebkha Salt Lake (Tunisia). *Afr. J. Microbiol. Res.* 8 355–367. 10.5897/AJMR12.1087

[B21] Houchstein L. I. Dalton B. P. (1968). Factors affecting the cation requirement of a halophilic NADH dehydrogenase. *Biochim. Biophys. Acta Enzymol.* 167 638–640. 10.1016/0005-2744(68)90062-4 4301885

[B22] Hynek B. M. Osterloo M. K. Kierein-Young K. S. (2015). Late-stage formation of Martian chloride salts through ponding and evaporation. *Geology* 43 787–790. 10.1130/G36895.1

[B23] Javor B. J. (1984). Growth potential of halophilic bacteria isolated from solar salt environments: carbon sources and salt requirements. *Appl. Environ. Microbiol.* 48 352–360. 16346609 10.1128/aem.48.2.352-360.1984 PMC241517

[B24] Karan R. Capes M. D. Dassarma S. (2012). Function and biotechnology of extremophilic enzymes in low water activity. *Aquat. Biosyst.* 8:4. 10.1186/2046-9063-8-4 22480329 PMC3310334

[B25] Kargel J. (2000). Europa’s crust and ocean: origin, composition, and the prospects for life. *Icarus* 148 226–265. 10.1006/icar.2000.6471

[B26] Lanyi J. K. (1974). Salt-dependent properties of proteins from extremely halophilic bacteria. *Bacteriol. Rev.* 38 272–290. 4607500 10.1128/br.38.3.272-290.1974 PMC413857

[B27] Li W. J. Schumann P. Zhang Y. Q. Chen G. Z. Tian X. P. Xu L. H. (2005). *Marinococcus halotolerans* sp. nov., isolated from Qinghai, north-west China. *Int. J. Syst. Evol. Microbiol.* 55 1801–1804. 10.1099/ijs.0.63596-0 16166669

[B28] Lindemann S. R. Moran J. J. Stegen J. C. Renslow R. S. Hutchison J. R. Cole J. K. (2013). The epsomitic phototrophic microbial mat of Hot Lake, Washington: community structural responses to seasonal cycling. *Front. Microbiol.* 4:323. 10.3389/fmicb.2013.00323 24312082 PMC3826063

[B29] Louis P. Galinski E. A. (1997). Characterization of genes for the biosynthesis of the compatible solute ectoine from *Marinococcus halophilus* and osmoreg u lated expression in *Escherichia coli*. *Microbiology* 143 1141–1141. 10.1099/00221287-143-4-1141 9141677

[B30] Madern D. Ebel C. Zaccai G. (2000). Halophilic adaptation of enzymes. *Extremophiles* 4 91–98. 10.1007/s007920050142 10805563

[B31] Mcgenity T. J. Oren A. (2012). “Hypersaline environments and halophiles,” in *Life at Extremes: Environments, Organisms and Strategies for Survival* ed. Bell E. M. (Wallingford: CAB International) 402–437. 10.1079/9781845938147.0402

[B32] Novitsky T. J. Kushner D. J. (1976). *Planococcus halophilus* sp. nov., a facultatively halophilic coccus. *Int. J. Syst. Bacteriol.* 26 53–57. 10.1099/00207713-26-1-53

[B33] Oren A. (2011). Thermodynamic limits to microbial life at high salt concentrations. *Environ. Microbiol.* 13 1908–1923. 10.1111/j.1462-2920.2010.02365.x 21054738

[B34] Oren A. Larimer F. Richardson P. Lapidus A. Csonka L. N. (2005). How to be moderately halophilic with broad salt tolerance: clues from the genome of *Chromohalobacter salexigens*. *Extremophiles* 9 275–279. 10.1007/s00792-005-0442-7 15902510

[B35] Ortega G. Laín A. Tadeo X. López-Méndez B. Castaño D. Millet O. (2011). Halophilic enzyme activation induced by salts. *Sci. Rep.* 1:6. 10.1038/srep00006 22355525 PMC3216494

[B36] Pontefract A. Zhu T. F. Walker V. K. Hepburn H. Lui C. Zuber M. T. (2017). Microbial diversity in a hypersaline sulfate lake: a terrestrial analog of ancient Mars. *Front. Microbiol.* 8:1819. 10.3389/fmicb.2017.01819 29018418 PMC5623196

[B37] Renaut R. W. (1990). “Recent carbonate sedimentation and brine evolution in the saline lake basins of the Cariboo Plateau, British Columbia, Canada,” in Saline Lakes. *Developments in Hydrobiology* Vol. 59 eds Comín F. A. Northcote T. G. (Dordrecht: Springer) 67–81. 10.1007/978-94-009-0603-7_6

[B38] Rice M. S. Bell J. F. III Cloutis E. A. Wang A. Ruff S. W. Craig M. A. (2009). Silica-rich deposits and hydrated minerals at Gusev Crater, Mars: Vis-NIR spectral characterization and regional mapping. *Icarus* 205 375–395. 10.1016/j.icarus.2009.03.035

[B39] Saitou N. Nei M. (1987). The neighbor-joining method: a new method for reconstructing phylogenetic trees’. *Mol. Biol. Evol.* 4 406–425. 3447015 10.1093/oxfordjournals.molbev.a040454

[B40] Soppa J. (2006). From genomes to function: haloarchaea as model organisms. *Microbiology* 152 585–590. 10.1099/mic.0.28504-0 16514139

[B41] Stevenson A. Burkhardt J. Cockell C. S. Cray J. A. Dijksterhuis J. Fox-Powell M. (2015). Multiplication of microbes below 0.690 water activity: Implications for terrestrial and extraterrestrial life. *Environ. Microbiol.* 17 257–277. 10.1111/1462-2920.12598 25142751

[B42] Tadeo X. López-Méndez B. Trigueros T. Laín A. Castaño D. Millet O. (2009). Structural basis for the aminoacid composition of proteins from halophilic archea. *PLoS Biol.* 7:e1000257. 10.1371/journal.pbio.1000257 20016684 PMC2780699

[B43] Tosca N. J. Knoll A. H. McLennan S. M. (2008). Water activity and the challenge for life on early Mars. *Science* 320 1204–1207. 10.1126/science.1155432 18511686

[B44] Tosca N. J. McLennan S. M. Lamb M. P. Grotzinger J. P. (2011). Physicochemical properties of concentrated Martian surface waters. *J. Geophys. Res.* 116 1–16. 10.1029/2010JE003700

[B45] Urakawa H. Martens-Habbena W. Stahl D. A. (2010). High abundance of ammonia-oxidizing archaea in coastal waters, determined using a modified DNA extraction method. *Appl. Environ. Microbiol.* 76 2129–2135. 10.1128/AEM.02692-09 20118363 PMC2849251

[B46] Van Hao M. Kocur M. Komagata K. Komagata K. (1984). *Marinococcus* gen. nov., A new genus for motile cocci with meso-diaminopimelic acid in the cell wall; and *Marinococcus albus* sp. nov. and *Marinococcus halophilus* (Novitsky and Kushner) comb. nov. *J. Gen. Appl. Microbiol.* 30 449–459. 10.2323/jgam.30.449

[B47] Vaniman D. T. Bish D. L. Chipera S. J. Fialips C. I. Carey J. W. Feldman W. C. (2004). Magnesium sulphate salts and the history of water on Mars. *Nature* 431 663–665. 10.1038/nature02973 15470421

[B48] Ventosa A. Nieto J. J. Oren A. (1998). Biology of moderately halophilic aerobic bacteria. *Microbiol. Mol. Biol. Rev.* 62 504–544. 9618450 10.1128/mmbr.62.2.504-544.1998 PMC98923

[B49] Wallmann K. Aghib F. Castradori D. Cita M. Suess E. Greinert J. (2002). Sedimentation and formation of secondary minerals in the hypersaline Discovery Basin, eastern Mediterranean. *Mar. Geol.* 186 9–28. 10.1016/S0025-3227(02)00170-6

[B50] Wang Y. Cao L. L. Tang S. K. Lou K. Mao P. H. Jin X. (2009). *Marinococcus luteus* sp. nov., a halotolerant bacterium isolated from a salt lake, and emended description of the genus *Marinococcus*. *Int. J. Syst. Evol. Microbiol.* 59 2875–2879. 10.1099/ijs.0.009670-0 19628611

[B51] Winston P. W. Bates D. H. (1960). Saturated solutions for the control of humidity in biological research. *Ecology* 41 232–237. 10.2307/1931961

[B52] Yakimov M. M. La Cono V. Spada G. L. Bortoluzzi G. Messina E. Smedile F. (2015). Microbial community of the deep-sea brine Lake Kryos seawater-brine interface is active below the chaotropicity limit of life as revealed by recovery of mRNA. *Environ. Microbiol.* 17 364–382. 10.1111/1462-2920.12587 25622758

[B53] Yen A. S. Gellert R. Schröder C. Morris R. V. Bell J. F. III Knudson A. T. (2005). An integrated view of the chemistry and mineralogy of Martian soils. *Nature* 436 49–54. 10.1038/nature03637 16001059

